# Oncological outcome of complete response after neoadjuvant chemotherapy for breast conserving surgery: a systematic review and meta-analysis

**DOI:** 10.1186/s12957-017-1273-6

**Published:** 2017-11-28

**Authors:** Xuan Li, Danian Dai, Bo Chen, Hailin Tang, Weidong Wei

**Affiliations:** Present Address: Department of Breast Oncology, Sun Yat-Sen University Cancer Center, 651 East Dongfeng Road, Guangzhou, 510060 China

**Keywords:** Neoadjuvant, Chemotherapy, Breast cancer, Chemotherapy response, Predictive value

## Abstract

**Background:**

With limited sample sizes and single-institution designs, how complete response (CR) after neoadjuvant chemotherapy (NAC) influences breast conserving surgery (BCS) and its value in prognosis are not clear.

**Methods:**

A systematic research review was conducted using electronic database. The rate of clinical complete response (cCR) in BCS after NAC and these pathological CR (PCR) and non-pCR BCS patients’ local recurrence-free survival (LRFS), distance recurrence-free survival (DRFS), overall survival (OS), and disease-free survival (DFS) rates were collected. A pooled analysis was performed using a fixed or random effects model and a *Q* test to determine heterogeneity.

**Results:**

Sixteen studies with a total of 4639 patients were included. The pooled data revealed that cCR patients compared with non-cCR patients had significantly higher rates of BCS, with a summary estimate odds ratios (OR) of 4.54 (95% CI 2.03–10.17). The pooled data revealed that BCS patients who achieved pCR after NAC had significantly lower rates of LRFS (RR = 0.59, 95% CI 0.38–0.92) and DRFS (RR = 0.27, 95% CI 0.13–0.55). Better DFS (RR = 0.09, 95% CI 0.04–0.25) and OS (RR = 0.36, 95% CI 0.03–3.90) were also seen, but OS was not significantly different.

**Conclusions:**

The rate of successful BCS is higher in the cCR group than in the non-cCR group, means cCR after NAC can encourage patients to receive BCS. The achievement of pCR after NAC in BCS patients was associated with a good prognosis in terms of LRFS and DRFS, but its value in DFS and OS requires further investigation.

**Electronic supplementary material:**

The online version of this article (10.1186/s12957-017-1273-6) contains supplementary material, which is available to authorized users.

## Background

Breast cancer is known as the most common cancer with the second highest mortality in females [1]. Recently, the use of NAC has been extended to downstage tumors and enhances the BCS rate [2–4]. Some studies have reported equal survival benefits between preoperative and postoperative chemotherapy [5–7], and with the gradual improvement of postoperative radiotherapy after BCS, BCS after NAC has become more and more accepted.

The survival benefit for patients who achieve pCR after NAC is controversial. Some studies found that pCR patients have a better prognosis than non-pCR patients but Cortazar P put out in his meta-analysis that in trial-level studies, the association between pCR and both event-free survival (EFS) and OS was weak [8, 9]. Recurrence following BCS after NAC is concerned by patients and doctors. Some studies reported that BCS after NCT leads to loco-regional recurrence (LR) rates of less than 10%, but other studies reported LR rates greater than 20% [10–13]. These conclusions were drawn from studies in which the patients were not carefully separated into BCS and mastectomy treatment (MT) groups or according to CR and non-CR.

How CR influences BCS is unclear because of limited high-quality data. For these reasons, we collected data on diagnosis, treatment, and prognosis from patients who received BCS after NAC and pooled the results for analysis in this study. The goal was to explore the rate of BCS in cCR compared to non-cCR patients after NAC and if other factors influenced this outcome. We also investigated whether pCR is a prognostic factor in BCS patients relative to LRFS, DRFS, DFS, and OS compared with BCS patients who did not achieve pCR. The potential prognostic value of pCR status may be very useful in BCS patients who received NAC because it can help in the determination of follow-up treatments and monitoring.

## Methods

### Literature search

**Fig. 1:**
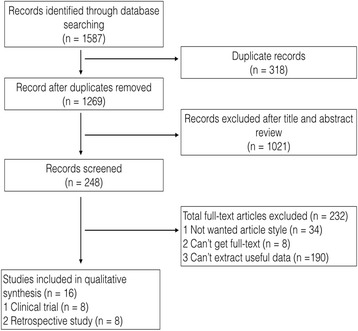
Flow chart of study selection process

We carried out a systematic literature search using PubMed, Embase, and the Web of Science through June 20, 2017. The search headings were as follows: (breast conserving surgery OR breast conserving operation OR lumpectomy OR quadrantectomy) AND (event-free survival OR overall survival OR disease-free survival OR DFS OR OS OR treatment outcome) AND (exp breast neoplasms OR cancer OR neoplasm OR carcinoma OR exp. neoadjuvant therapy OR neoadjuvant OR exp. preoperative care) AND (cohort OR exp. cohort studies OR longitudinal OR prospective OR retrospective OR exp. clinical trial OR double-blind method OR clinical trial OR randomized controlled trial OR multicenter study OR exp. clinical trials as topic OR controlled trial OR single OR double trial OR blind trail). In all, we found 1587 articles, including 247 from PubMed, 865 from the Web of Science, and 475 from Embase (Fig. 1).

### Study selection criteria

The published studies to be included in this analysis met the following criteria: (1) a focus on breast cancer patients who were treated with neoadjuvant chemotherapy and then received surgery, (2) the presence of evaluations of the associations between CR and outcomes in patients who received either breast conserving surgery or mastectomy surgery, (3) the presence of the necessary information to calculate outcomes (LRFS, DRFS, DFS, or OS) of interest, and (4) availability as a full-text English-language publication. When multiple publications from the same author or institution appeared that presented duplicate data, we chose the most applicable one. Systematic reviews, meta-analyses, case studies, and articles that did not meet our terms were excluded.

### Data extraction

The following information was extracted from each eligible study: the first authors’ names, the country in which the data were collected, the publication year, the applied neoadjuvant chemotherapy regimens, the total number of patients, mean age, tumor size, follow-up time, the number of patients who received BCS, the number patients who achieved CR, and the survival data (percentages and numbers of events) of BCS patients who achieved CR and those who did not. Other information was also recorded if the author found it useful. We used The Newcastle-Ottawa Scale (NOS) to assess the quality of each study by two authors. The scores included three parts: the selectivity of patients (0–4), comparability of groups (0–2) and assessment of outcome (0–3). Scores > 5 were considered high-quality studies.

### Statistical analysis

A meta-analysis was conducted for each endpoint of interest when there were two or more studies available. The data were pooled with the fixed-effects model by default, but if homogeneity across the tests was identified by the *Q* statistic and the *I* ^2^ value presented significant homogeneity as *I* ^2^ > 50% or Cochran *Q* < 0.1, a random effects model was used. If significant heterogeneity appeared, a subgroup analysis according to neoadjuvant chemotherapy type and a sensitivity analysis to exclude unsuitable studies were used to determine the source of heterogeneity.

Using the data on the extent to which CR influences BCS conduct, we analyzed each study and calculated pooled odds ratios (OR) and 95% CIs. LRFS, DRFS, DFS, and OS were compared between the CR group and non-CR group of BCS patients using risk ratios (RR) and 95% CIs. A 95% CI that did not include zero was considered significantly different. A power analysis was performed for each study, and the pooled results were evaluated using Power and Precision software following the manufacturer’s instructions.

We investigated whether funnel plots presented symmetry to generally assess the publication bias. All the collected data were analyzed using RevMan 5.3 analysis software (Cochrane Collaboration, Copenhagen, Denmark), and all statistical tests were two-sided with statistical significance defined as *P <* 0.05.

## Results

We identified 1587 records, and through careful assessments, 16 studies were identified as eligible for the data abstract [10, 14–28]. Two studies were from the same author, but the data abstracts were different, so we included both studies [10, 14]. Among these 16 studies, five were random clinical trials, three were non-random clinical trials, and eight were retrospective cohort studies. Under the NOS quality system, all the studies’ score greater than five so the quality of the date met the analysis requirement. The details of the 16 studies included in the meta-analysis are reported in Table 1. The specific number and power values of the studies are shown in Table 2. Seven papers reported cCR data, and the others all reported pCR data. According to each author’s definition, four studies defined pCR as the absence of an invasive component in the primary breast tumor, regardless of pathological axillary node status, and the others defined pCR as the absence of an invasive component in both the breast and the axillary node. Among these reports with abstract data on pCR and non-pCR in BCS patients, 11 studies provided LRFS values for pCR and non-pCR individually, four studies provided DRFS values, and only two studies provided DFS and OS values. The power to detect OS was low, only 0.589. In contrast, the pooled power was greater than 0.9 for LRFS, DRFS, and DFS.

**Table 1:** Baseline characteristics and quality assessment of included studies

	General information								NOS score			
Author	Study design	Publish year	Country of enrolment	Neoadjuvant chemotherapy protocal	Total patients	Mean age (year)	Tumor size	Follow-up time (month)	Selection	Comparability	Outcome	Total score
Beriwal [24]	RS	2006	USA	CMF or AC or CAF or ACT in unclear cycle and interval time	153	52	T I,II,III,IV	60	3	1	2	6
Bonadonna [20]	CT	2002	Italy	Single-agent E 3 cycles every 3 weeks	317	49	T II,III	60	3	2	2	7
Caudle [19]	RS	2012	USA	Anthracycline-based or taxane-based or a combination of the two	595	51	T I,II,III,IV	64	3	1	2	6
Cho [22]	RS	2013	Korea	Unclear	431	49	T IV	46	2	1	2	5
Criscitiello [28]	RCT	2013	Spain	Paclitaxel with HER2-targeted therapy	455	NA	T II,III,IV	NA	3	2	2	7
Fastner [17]	RCT	2014	UAS	ED-based 3–6 cycles	107	48	T II,III	59	4	2	3	9
Jimbo [21]	RS	2015	Japan	4 cycles of AC or FEC followed by weekly T	315	50	T I,II,III,IV	61	3	1	2	6
Massidda [15]	CT	2007	Italy	PEV 6 cycles every 2 weeks	40	48	T IV	84	3	1	2	6
McIntosh [18]	RS	2003	England	CVAP* 4 or 6 cycles every 3 weeks	166	51	T II,III,IV	62	2	1	2	5
Noh [25]	RS	2014	Korea	AT or AC or T	260	46	T I,II,III,IV	66	2	1	2	5
Rouzier [10]	RS	2001	France	FAC or CMF or CTF or CE 4 cycles every 4 weeks	257	47	T I,II,III	93	3	1	2	6
Rouzier [14]	RCT	2004	France	Neoadjuvant epirubicin-based chemotherapy(AVCMF, FEC, FAC) 3–4 cycles at 21-day intervals or longer	589	50	T II,III	67	3	2	3	8
Shen [16]	RCT	2004	USA	Doxorubicin-based and paclitaxel-based	33	52	T IV	91	3	2	3	8
Von [27]	RCT	2008	Germany	TAC*6 or 8 cycles if response. TAC*2 if response then followed by another TAC 4 or 6 cycles, if not response then followed by NX or TAC 4 cycles	622	51.8	T I,II,III,IV	33	4	2	3	9
Walker [23]	CT	2011	England	AC 4 cycles every 3 weeks followed by weekly or every 3 weeks docetaxel	82	50	T II,III,IV	72	3	2	3	8
Yamazaki [26]	RS	2015	Japan	AT-based 4 cycles	217	52	T I,II,III,IV	84	3	1	2	6

*Abbreviation*: *RS* retrospective study, *RCT* randomized clinical trial, *CT* clinical trial, *A* doxorubicin, *T* taxane-based drug, *E* epirubicin, *D* docetaxel, *C* cyclophoshamide, *F* 5-fluorouracil, *M* methotrexate, *P* cisplatin, *V* vincristine, P* prednisolone, *X* capecitabine, *N* vinorelbine, *NA* not available, *NOS* Newcastle-Ottawa Scale

**Table 2:** RR and study power in LRFS, DRFS, DFS, and OS in BCS

Study	pCR, *n* (%)	LR			DR			DFS			OS		
		Overall event/event	RR	Power	Overall event/event	RR	Power	Overall event/event	RR	Power	Overall event/event	RR	Power
Caudle 2012 [19]	124 (20.8)	37/3	0.34	0.605	NA	NA	NA	NA	NA	NA	NA	NA	NA
Bonadonna 2002 [20]	7 (3.3)	8/0	1.52	0.106	NA	NA	NA	NA	NA	NA	NA	NA	NA
Fastner 2014 [17]	15 (14.0)	6/0	0.45	0.247	NA	NA	NA	NA	NA	NA	NA	NA	NA
Noh 2014 [25]^a^	102 (39.2)	13/2	0.28	0.476	18/1	0.09	0.911	55/3	0.09	1	9/0	0.08	0.751
Shen 2004 [16]^a^	4 (12.1)	5/0	0.55	0.195	9/0	0.32	0.345	NA	NA	NA	NA	NA	NA
Cho 2013 [22]	38 (30.6)	6/0	0.17	0.482	16/1	0.15	0.723	21/1	0.11	0.897	15/4	0.82	0.066
Jimbo 2014 [21]	63 (32.5)	5/2	1.39	0.065	NA	NA	NA	NA	NA	NA	NA	NA	NA
Yamazaki 2015 [26]^a^	56 (25.8)	14/6	2.16	0.276	NA	NA	NA	NA	NA	NA	NA	NA	NA
Rouzier 2001 [10]	28 (10.9)	41/2	0.42	0.328	71/4	0.49	0.441	NA	NA	NA	NA	NA	NA
Mclntosh 2003 [18]	18 (40.9)	1/0	0.47	0.138	NA	NA	NA	NA	NA	NA	NA	NA	NA
Beriwal 2006 [24]^a^	37 (24.2)	21/4	0.74	0.095	NA	NA	NA	NA	NA	NA	NA	NA	NA
total	492 (22.4)	157/19	0.59	0.933	114/6	0.27	1	76/4	0.09	1	24/4	0.36	0.589

*Abbreviation*: *RR* risk ratio, *LR* local recurrence, *DR* distance recurrence, *DFS* disease-free survival, *OS* overall survival, *pCR* pathology complete response, *NA* not available

^a^Means the definition of pCR was the absence of invasive component in the primary breast tumor, irrespective of pathological axillary node status

### Primary endpoint: cCR and its effect on BCS conduct

**Fig. 2:**
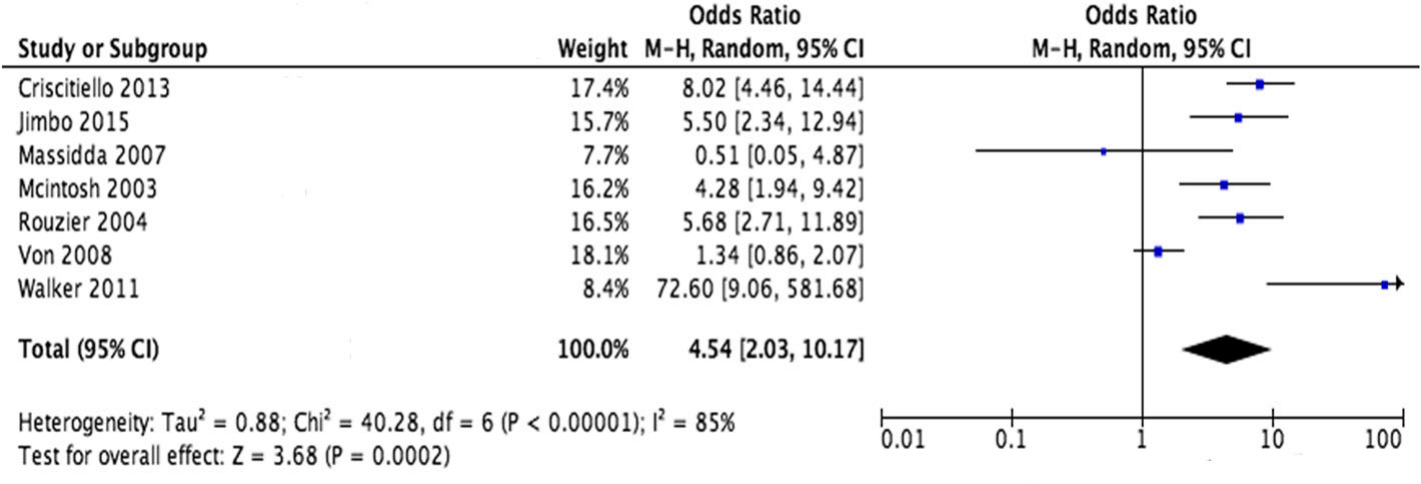
Forest plots showing OR and the 95% CI for cCR compared with non-cCR in BCS conduct rate. Note: *OR* odds ratios, *cCR* clinical complete response

Seven studies reported exact data of BCS rate in cCR and non-cCR groups. About 352 patients gained cCR, among them, 213 patients received BCS; in 1713 patients not gained cCR, about 624 patients received BCS. The pooled data revealed that cCR patients compared with non-cCR patients had significantly higher rates of BCS and the summary estimate OR was 4.54 (95% CI 2.03–10.17). Heterogeneity testing revealed *I* ^2^ = 85% with *P* < 0.001 (random effects model; Fig. 2). We then divided these studies into two groups according to neoadjuvant chemotherapy type. Neoadjuvant chemotherapy in group 1 included a taxane-anthracycline-based protocol and group 2 included an anthracycline without taxane protocol. Group 1 had the higher BCS rate in cCR patients (OR = 6.25, 95% CI 1.74–22.39) than group 2 (OR = 3.81, 95% CI 1.65–8.78), but the two groups did not show subgroup significant heterogeneity (*I* ^2^ = 0% with *P* = 0.53, Additional file 1: Figure S1a).

### Secondary endpoints: LR and DR rates in BCS patients who did or did not achieve pCR

**Fig. 3:**
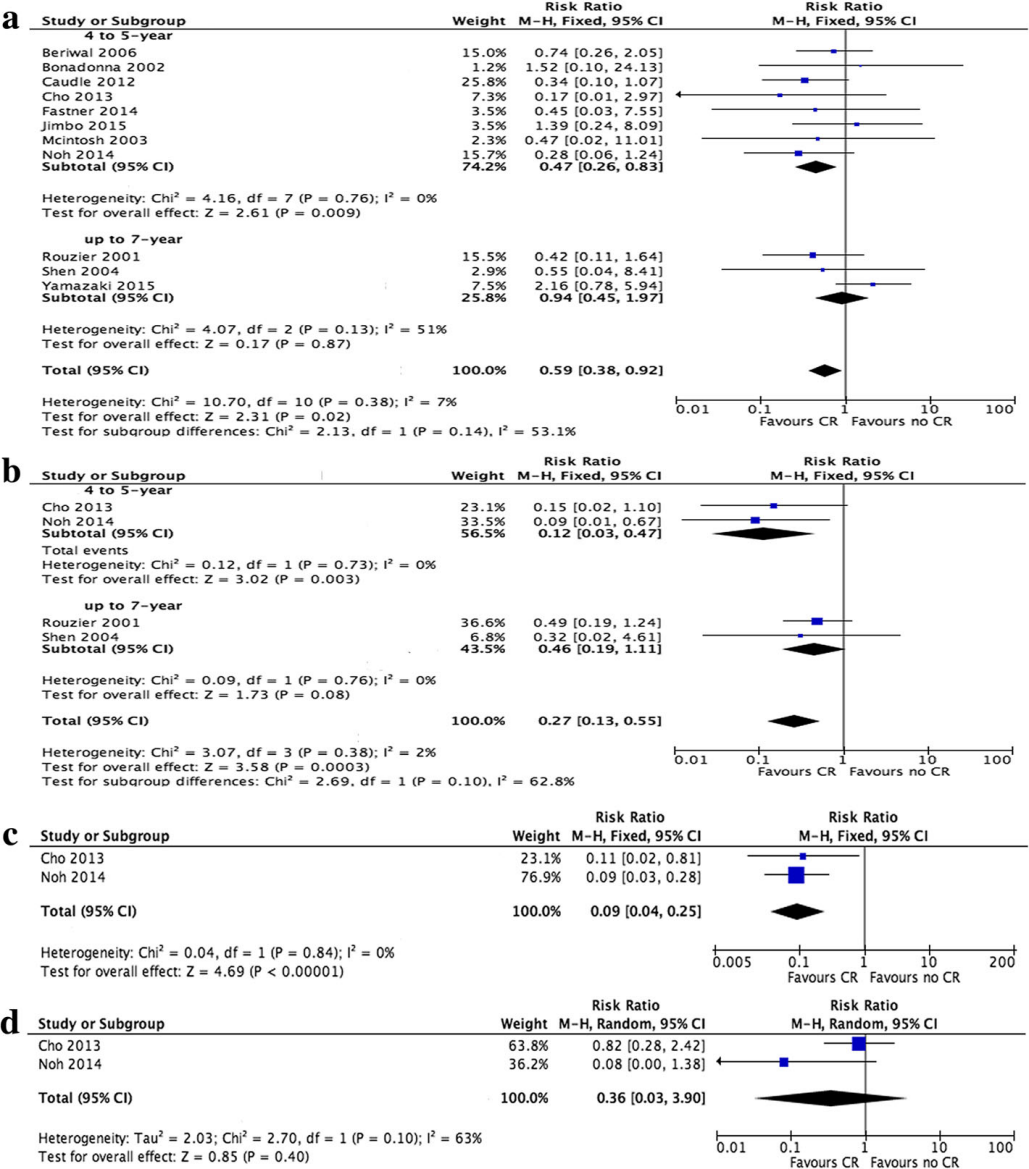
Forest plots showing RR and the 95% CI of LRFS (**a**), DLFS (**b**), DFS (**c**), and OS (**d**) for pCR vs. non-pCR in BCS. Note: *BCS* breast conserving surgery, *LRFS* local recurrence-free survival, *DRFS* distant recurrence-free survival, *OS* overall survival, *DFS* disease-free survival, *pCR* pathology complete response, *RR* risk ratios, *DR* distant recurrence

Eleven studies reported LR rates in BCS patients (2197 patients). In BCS patients, the overall LR rate was 3.9% (range 0–10.8%) in pCR patients and 8.1% (range 2.3–17.2%) in non-pCR patients. The pooled data revealed that the BCS patients who achieved pCR after neoadjuvant chemotherapy had significantly lower rates of LR, with a summary estimate RR of 0.59 (95% CI 0.38–0.92). Heterogeneity testing revealed *I* ^2^ = 7% with *P* = 0.38 (fixed effects model, Fig. 3a). Subgroup analysis showed that BCS patients with pCR had statistic significant differences better LRFS (RR = 0.47, 95% CI 0.26–0.83) in 5-year follow-up while not show statistic significant differences in 7-year follow-up (RR = 0.94, 95% CI 0.45–1.97, Fig. 3a**)**.

Four studies reported DR rates in BCS patients (674 patients). In BSC patients, the overall DR rate was 3.5% (varied 0–14.3%) in pCR patients and 21.5% (varied 10.8–31.0%) in non-pCR patients. The pooled data revealed that BCS patients with pCR had significantly lower DR rates, with a summary estimate RR of 0.27 (95% CI 0.13–0.55). Heterogeneity testing revealed *I* ^2^ = 2% with *P* = 0.38 (fixed effects model, Fig. 3b). In subgroup analysis, BCS patients with pCR in 5 year follow-up had significant difference in DRFS (RR = 0.12, 95% CI 0.03–0.47) while in a 7-year follow-up had no significant difference (RR = 0.46, 95% CI 0.19–1.11, Fig. 3b).

Two studies that reported LR data and another two studies that reported both LR and DR data were excluded by the sensitivity analysis because of the pCR definition they used, which was the absence of an invasive component in the primary breast tumor instead of in both the breast and the axillary node. However, the advantage of pCR in BCS remained significantly different, with an RR of 0.44 (95% CI 0.22–0.86, heterogeneity testing *I* ^2^ = 0% with *P* = 0.80, Additional file 1: Figure S1b) in LRFS. The RR was 0.36 (95% CI 0.16–0.82, heterogeneity testing *I* ^2^ = 13% with *P* = 0.28, Additional file 1: Figure S1c) in DRFS.

### Tertiary endpoints: DFS and OS in BCS patients who did and did not achieve pCR

Only two studies provided DFS and OS rates in pCR and non-pCR patients who received BCS. The median follow-up times were 66 and 46 months. We evaluated these two studies in the meta-analysis and found that DFS rates were higher in BCS patients with pCR, with a summary estimate RR of 0.09 (95% CI 0.04–0.25, heterogeneity testing *I* ^2^ = 0% with *P* = 0.84, Fig. 3c). OS was higher in pCR patients, but without a significant difference, as the RR was 0.36 (95% CI 0.03–3.90, heterogeneity testing *I* ^2^ = 63% *P* = 0.10, Fig. 3d).

## Publication bias

**Fig. 4:**
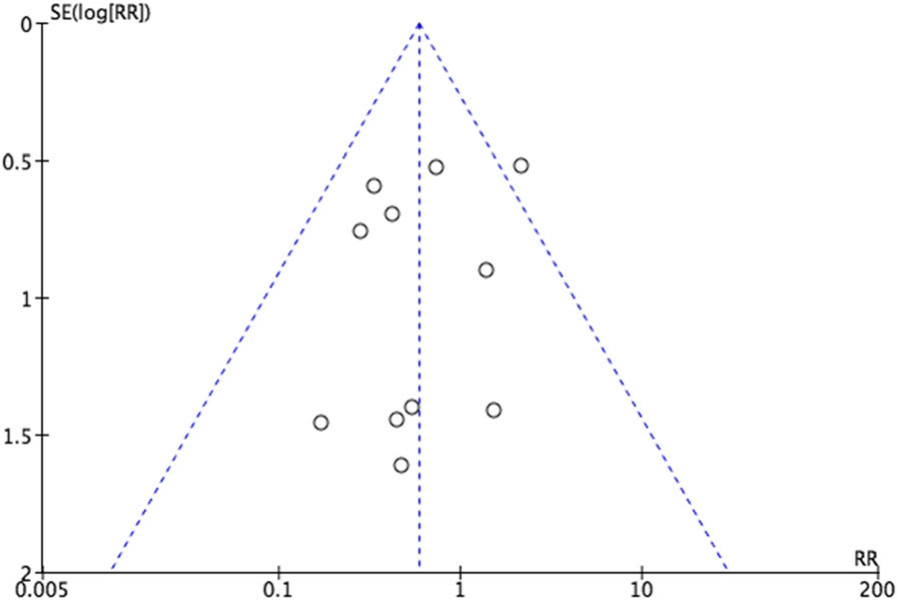
Funnel plots of RR pCR compared with non-pCR in BCS conduct rate with the standard error (SE). Vertical line represents the pooled effect estimate. Note: *RR* risk ratios, *pCR* pathology complete response, *BCS* breast conserving surgery

The publication bias was evaluated with funnel plots. The funnel plots showed no obvious evidence for a publication bias as the plot was basically inverted and funnel-shaped with bilateral symmetry (Fig. 4).

## Discussion

Recently, NAC has been widely used in breast cancer patients to downstage tumor size, increase the rate of resection, or increase eligibility for breast conserving surgery [29, 30]. BCS success and its prognosis were influenced by many traditional factors such as pathological features, primary tumor size, lymph node metastasis, and margin status. Patients who receive NAC exhibit one of four responses to cancer, including complete response (CR), partial response (PR), stable disease (SD), and progressive disease (PD). Therefore, in-patients who received NAC and then BCS, whether CR can affect the operation rate of BCS and whether CR can be used as an accurate prediction marker, were discussed in this meta-analysis.

BCS rates reportedly increased overall after NAC [31]. In our study, we analyzed patients with and without cCR who received BCS and found that BCS was more likely to be successful in cCR patients. Of the seven studies included in this meta-analysis, we found significant statistical heterogeneity. NAC regimens in our study were classified as paclitaxel-epirubicin-based regimen and epirubicin without paclitaxel-based regimen. Paclitaxel-epirubicin-based regimen had higher BCS rate, and this may attribute to its superior downstaging effects [32].

Higher response and safe of BCS after tumor downstage can inspire more patients to receive BCS [33].

LRFS and DRFS were not significantly different between the BCS and MT groups [34, 35] as well as like some studies said, following neoadjuvant chemotherapy [36, 37]. However, some researchers found that BCS patients had higher LR after NAC, perhaps because the surgical margins became difficult to accurately assess [38], some studies reported contrary results that MT group had higher LR rate compared with BCS group [18, 39]. In an NSABP-B18 trial, a higher rate of LR was found in the NCT-BCS group compared to the control group, but after adjusting for tumor size and patient age, the difference was no longer significant [40]. The achievement of pCR after NAC seems to indicate a good prognosis for operable patients [41, 42], but in trial-level studies, this parameter was not recommended in the determination of EFS or OS [43]. The studies included in our analysis should be carefully compared because they used different definitions of pCR. Some studies focused on responses in the breast only, regardless of nodal involvement (ypT0/ypNx), and some used more stringent definitions that involved no residual tumor in either the breast or the node (ypT0/ypN0). We excluded studies in which only breast responses were evaluated and found that pCR patients who received BCS still had lower LR and DR rates compared with non-pCR patients who received BCS. In patients with longer follow-up time, as the included studies with limited numbers, the benefit of pCR in BCS patients’ local and distance control still in dispute. Therefore, the achievement of pCR may increase the safety of local and distant recurrence control measures in BCS patients, but how to select pCR patients before surgery needs more research such as tumor markers and accurate imaging tools.

In our study, the DFS rates were significantly higher in BCS patients with pCR. OS was higher in BCS patients with pCR, but no significant differences were observed. In another meta-analysis that included BCS and MT patients, pCR achievement following NAC indicated a good prognosis in terms of DFS and OS [42, 44]. Because of our limited data and follow-up time, the results of our study relative to DFS and OS require more data for credibility.

There are some limitations in our study. First, this analysis relied on a slightly different definition of pCR. Although we separately analyzed pCR only in the breast, pCR definition was still somewhat different compared to some studies that included in situ contents and some that did not. Second, adjuvant therapy and BCS methods including lumpectomy or quadrantectomy with sentinel lymph node biopsy or axillary dissection were mixed and matched according to the judgment of the doctor, without a united protocol. Almost all patients who received BCS also received radiotherapy, but different doses, regions, and methods were used. Adjuvant chemotherapy was administered to some patients, especially non-pCR patients. More effective treatments were used in non-pCR patients, and this may offset the advantage of pCR values in BCS eligibility. Third, the data in this study were limited. We calculated the power of each study and the pooled power, and all values were higher than 0.8, except for the data on OS. Less data was available on OS, and the follow-up times were short, so the acceptance of these results warrants caution and critical thinking.

## Conclusions

Successful conduct rate of BCS was higher in the cCR group than in the non-cCR group; however, the relationship of cCR and pCR needs more research. BCS patients who achieved pCR after NAC demonstrated a good prognosis in terms of LRFS and DRFS; though also seem favors in DFS and OS, the value of pCR in long-time survival of BCS needs further investigation.

## Additional file

Additional file 1: Figure S1. Forest plots showing OR and the 95% CI for cCR compared with non-cCR in BCS groups. (**a**) Group 1 included a Paclitaxel-epirubicin-based taxane-anthracycline-based protocol, group 2 included an epirubicin without paclitaxel-basedanthracycline without taxane protocol; Forest plots showing RR and the 95% CI of LRFS (**b**), DLFS (**c**) removed four articles used definition of pCR as the absence of invasive component in the primary breast tumor instead of in both the breast and the axillary node for pCR vs. non-pCR in BCS. Note: BCS breast conserving surgery, LRFS local recurrence-free survival, DRFS distant recurrence-free survival, pCR pathology complete response, OR odds ratios, RR risk ratios. (TIFF 11982 kb)

## Abbreviations

BCS: Breast conserving surgery
cCR: Clinical complete response
CR: Complete response
DFS: Disease-free survival
DR: Distant recurrence
DRFS: Distant recurrence-free survival
EFS: Event-free survival
LR: Local recurrence
LRFS: Local recurrence-free survival
MT: Mastectomy
NAC: Neoadjuvant chemotherapy
OR: Odds ratios
OS: Overall survival
pCR: Pathology complete response
RR: Risk ratios
