# Supplementary figures and images for: Oncological outcome of complete response after neoadjuvant chemotherapy for breast conserving surgery: a systematic review and meta-analysis
Source: World J Surg Oncol. 2017 Nov 28;15:210. doi: 10.1186/s12957-017-1273-6 (PMC5706340; doi:10.1186/s12957-017-1273-6)

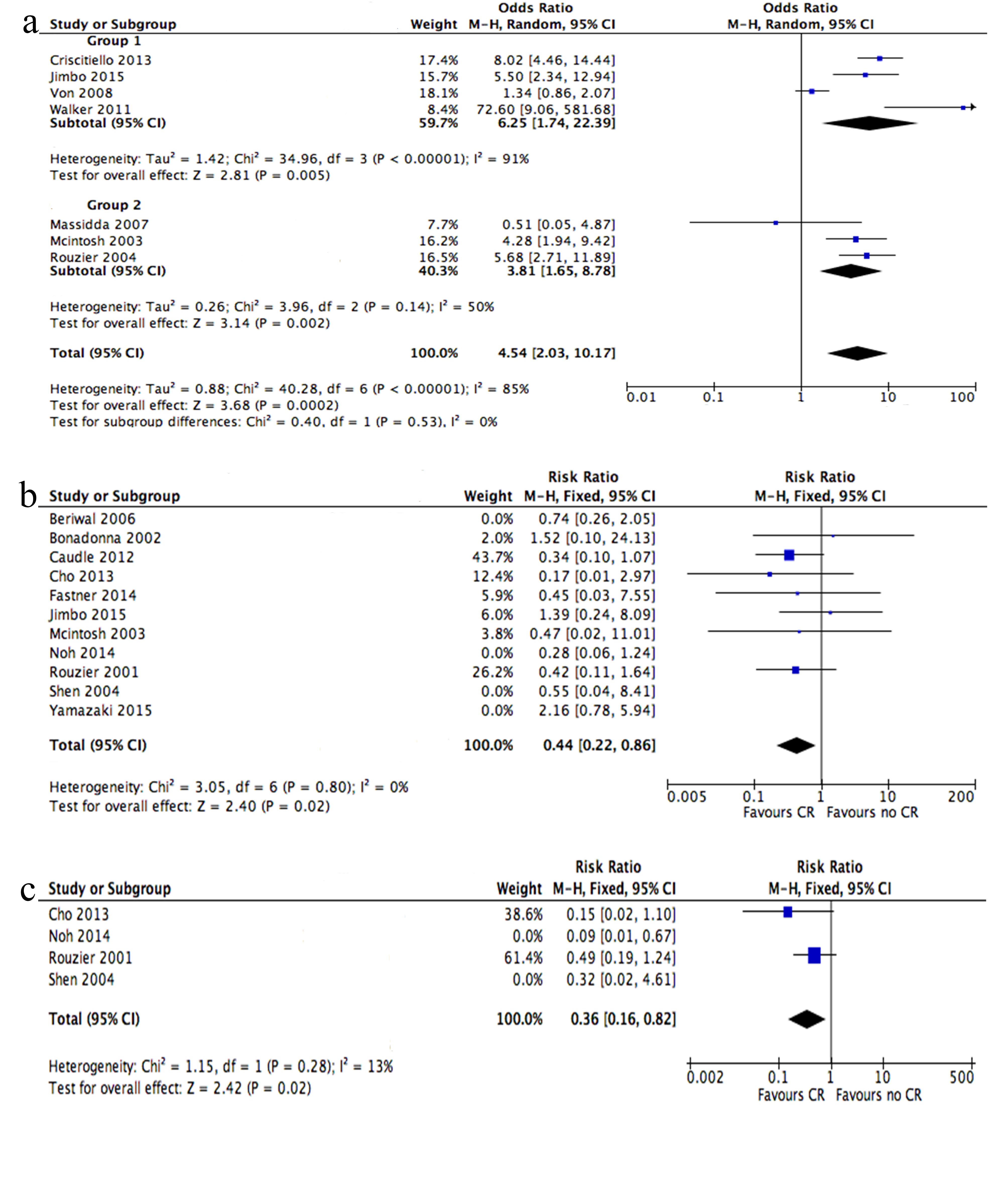

Supplement: Additional file 1: Figure S1. — Forest plots showing OR and the 95% CI for cCR compared with non-cCR in BCS groups. (a) Group 1 included a Paclitaxel-epirubicin-based taxane-anthracycline-based protocol, group 2 included an epirubicin without paclitaxel-basedanthracycline without taxane protocol; Forest plots showing RR and the 95% CI of LRFS (b), DLFS (c) removed four articles used definition of pCR as the absence of invasive component in the primary breast tumor instead of in both the breast and the axillary node for pCR vs. non-pCR in BCS. Note: BCS breast conserving surgery, LRFS local recurrence-free survival, DRFS distant recurrence-free survival, pCR pathology complete response, OR odds ratios, RR risk ratios. (TIFF 11982 kb) [file 12957_2017_1273_MOESM1_ESM.tif]
